# Lentinan inhibits tumor angiogenesis via interferon γ and in a T cell independent manner

**DOI:** 10.1186/s13046-018-0932-y

**Published:** 2018-10-29

**Authors:** Shengming Deng, Guoxi Zhang, Jiajie Kuai, Peng Fan, Xuexiang Wang, Pei Zhou, Dan Yang, Xichen Zheng, Xiaomei Liu, Qunli Wu, Yuhui Huang

**Affiliations:** 1 Cyrus Tang Hematology Center, Collaborative Innovation Center of Hematology, State Key Laboratory of Radiation Medicine and Prevention, Soochow University, 199 Ren-Ai Road, Suzhou, 215123 Jiangsu China; 2 Nanjing Luye Pharmaceutical Co., Ltd, Nanjing, 210061 Jiangsu China; 3 Department of Traditional Chinese Medicine, Peking Union Medical College Hospital, Peking Union Medical College and Chinese Academy of Medical Sciences, No. 1 Shuaifuyuan, Dongcheng District, Beijing, 100730 China

**Keywords:** Lentinan, Interferon γ, Antiangiogenic therapy, Drug resistance, Lung cancer

## Abstract

**Background:**

Antiangiogenic agents are commonly used in lung and colon cancer treatments, however, rapid development of drug resistance limits their efficacy.

**Methods:**

Lentinan (LNT) is a biologically active compound extracted from *Lentinus edodes*. The effects of LNT on tumor angiogenesis were evaluated by immunohistochemistry in murine LAP0297 lung and CT26 colorectal tumor models. The impacts of LNT on immune cells and gene expression in tumor tissues were determined by flow cytometry, qPCR, and ELISA. Nude mice and IFNγ blockade were used to investigate the mechanism of LNT affecting on tumor angiogenesis. The data sets were compared using two-tailed student’s *t* tests or ANOVA.

**Results:**

We found that LNT inhibited tumor angiogenesis and the growth of lung and colon cancers. LNT treatments elevated the expression of angiostatic factors such as IFNγ and also increased tumor infiltration of IFNγ-expressing T cells and myeloid cells. Interestingly, IFNγ blockade, but not T cell deficiency, reversed the effects of LNT treatments on tumor blood vessels. Moreover, long-lasting LNT administration persistently suppressed tumor angiogenesis and inhibited tumor growth.

**Conclusions:**

LNT inhibits tumor angiogenesis by increasing IFNγ production and in a T cell-independent manner. Our findings suggest that LNT could be developed as a new antiangiogenic agent for long-term treatment of lung and colon cancers.

**Electronic supplementary material:**

The online version of this article (10.1186/s13046-018-0932-y) contains supplementary material, which is available to authorized users.

## Background

New blood vessels must be developed to nourish tumors that grow bigger than 2 mm^3^ in volume, and the tumor vasculature is also a necessity for a tumor’s ability to metastasis; angiogenesis is thus a hallmark of cancer [1, 2]. Important proangiogenic factors that are known to be involved in tumor angiogenesis include proteins of the vascular endothelial growth factor (VEGF) family, the platelet-derived growth factor (PDGF) family, the fibroblast growth factor (FGF) family, and placental growth factor (PlGF) [1, 3]. Therefore, the blockade of proangiogenic signaling pathways has been investigated and developed as an antiangiogenic therapy aiming to starve tumor cells to death [3–6].

Antiangiogenic therapies are currently in wide use against non-small cell lung cancer (NSCLC), colorectal cancer, and several other types of solid tumors [1, 3]. The first antiangiogenic agent approved for NSCLC was bevacizumab, a monoclonal antibody to VEGF-A. The combination of bevacizumab and chemotherapeutic agents, such as carboplatin and paclitaxel, improved both progression-free survival and overall survival in advanced NSCLC patients, compared with chemotherapy alone [4]. However, the clinical benefits from VEGF inhibitors are modest and transient, and are usually followed by the rapid emergence of drug resistance [1, 3, 6]. As compensatory proangiogenic factors, such as PDGF and FGF, play key roles in mediating the resistance to VEGF signaling blockade therapy, multiple targeted kinase inhibitors (TKIs) could circumvent the common problem of rapid drug resistance onset because they simultaneously block several signaling pathways [1, 3]. The development of multitargeted TKIs is expected to combat this resistance, however, there have been few convincingly studies in the clinic to date [1, 7]. Moreover, marginal survival benefits and significant toxicities associated with TKIs have limited enthusiasm for this therapeutic approach. Therefore, a new strategy to better control tumor angiogenesis remains much anticipated.

Several recent reports suggest that immune stimulation can also restrict tumor angiogenesis. Activated T cells have been shown to decrease tumor vessel density, inhibit tumor endothelial cell proliferation, and/or arrest tumor blood flow [8–12], suggesting immune stimulators might have the capability to inhibit tumor angiogenesis. Lentinan (LNT), a biologically active compound extracted from *Lentinus edodes* (*L. edodes*), is an immunopotentiator [13–15], and exhibits multiple biological activities, including immunomodulatory, antibacterial, antivirus, antitumor effects, and anti-inflammatory [14, 16–19]. Although the combination of LNT with TNP-470 (TNP), an angiogenic inhibitor, displayed antiangiogenic effects and promoted tumor cell apoptosis [20], whether LNT affects tumor angiogenesis remains unclear. In this study, we evaluated the effects of LNT on the tumor vasculature in LAP0297 lung carcinoma and CT26 colorectal carcinoma. LNT treatments significantly reduced tumor vascular function and inhibited tumor growth. Moreover, long-term LNT treatments continuously suppressed tumor angiogenesis and exhibited antitumor effects. Mechanically, LNT inhibited tumor angiogenesis via IFNγ up-regulation, which was associated with the accumulation of tumor-infiltrating myeloid cells. Thus, this study demonstrates the potential of LNT to be served as a novel antiangiogenic agent for long-term cancer treatments.

## Methods

### Materials and reagents

The Lentinan (LNT, 1 mg, 16100108), a gift from Nanjing Luye pharmaceutical Co., Ltd. (Nanjing, China), is provided as powder in a penicillin bottle. LNT was dissolved in saline (0.9% NaCl) right before in vivo administration.

### Tumor models

Female FVB mice (6–8 weeks old) were bred and maintained in the gnotobiotic laboratory animal center in Soochow University. Female BALB/c and nude mice (6–8 weeks old) were purchased from the Shanghai Laboratory Animal Center (Shanghai, China) and the Vital River Laboratories (Beijing, China), respectively. All of the mice were housed in the specific pathogen-free (SPF) condition. FVB and Balb/c mice were subcutaneous (*s.c.*) inoculated with 2 × 10^5^ cells of LAP0297 or CT26 on the right flanks, respectively. When tumors reached 4–5 mm in diameter, mice bearing tumors were randomly divided into appropriate groups and subjected to Lentinan or saline (0.9% NaCl) treatments. In the long-term treatment experiments, mice bearing tumors were euthanized when tumor volume exceeded 1000 mm^3^. Tumor volume (mm^3^) was estimated by using the following formula: tumor volume = (long axis) × (short axis)^2^ × π/6.

### Cell lines

The LAP0297 lung carcinoma cell line was generated by Dr. Peigen Huang at Massachusetts General Hospital (Boston, USA) [21]. The CT26 tumor cell line was purchased from the American Type Culture Collection. Tumor cells were cultured in Dulbecco’s modified Eagle’s medium (DMEM) (GIBCO) supplemented with 10% heat-inactivated fetal bovine serum (FBS) (GIBCO) and 1% penicillin and streptomycin (GIBCO) at 37 °C in a humid incubator containing 5% CO_2_. Cell cultures were frequently monitored for mycoplasma contamination, and only mycoplasma-negative cells were used for experiments.

### Tumor vascular function analysis

The function of tumor blood vessels was determined by analyzing the intensity of Hoechst 33342 (Sigma), as described previously [22]. Briefly, 5 min after intravenous injection of Hoechst 33342 (10 mg/kg), mice were systemically perfused with PBS, and the tumors were removed and fixed with 4% paraformaldehyde. This procedure labeled functional vessels with fluorescence of nucleus-bound Hoechst 33342. Mosaic images of tumors were taken using an Olympus FV1000 confocal laser-scanning microscope. A 20× objective acquired 640 × 640 μm tiles, and an automated stage scanned through the entire cross section of a tumor tissue. The imaged tiles were stitched into a final mosaic image using an Olympus software. Nonspecific nuclear staining (Sytox green from Molecular Probes) was used to counter-stain the slides. In each field, the intensities of CD31 and Hoechst 33342-positive areas were calculated using an Image-Pro plus software (version 6.0).

### Immunohistochemistry

Tumor tissue samples were fixed for 2–3 h in 4% paraformaldehyde, and then incubated with 30% sucrose in PBS overnight at 4 °C. The tissue samples were then embedded in optical coherence tomography (OCT) compound and stored at − 80 °C. Frozen sections (20 μm) were incubated with a primary rat anti-mouse CD31 antibody (endothelial cell marker, 1:100, Clone MEC13.3, Cat#: 550274, BD Biosciences) and a secondary Alexa Fluor 647 donkey anti-rat IgG antibody (1:200, Jackson ImmunoResearch) to stain endothelial cells. The slides were counter-stained for cell nuclei by Sytox Green (Molecular Probes). Fluorescent images were taken using an Olympus FV1000 confocal laser scanning microscopy. Four to six photographic areas, excluding the tumor periphery, were randomly taken from each tumor tissue (640 × 640 μm^2^ each). Mean fluorescence intensity (MFI) of CD31 positive and Hoechst 33342 stained areas were calculated using an Image-Pro plus software (version 6.0).

### Tube formation assays

Tube formation assay was used to evaluate the effect of LNT on endothelial cells as descried previously with minor modifications [23, 24]. Briefly, human umbilical vein endothelial cells (HUVECs) were cultured in DMEM medium containing 10% FBS. Growth factor reduced matrigel matrix (CORNING) was placed in a refrigerator (4 °C) overnight to thaw the matrigel. Plates (24-well) were coated with 100 μl/well of matrigel matrix and were incubated at 37 °C for 30 min to allow the gel to solidify. HUVECs (30,000 cells/well) were seeded onto the top of the gel and were treated with different concentrations of LNT for 12 h in triplicate at 37 °C with 5% CO_2_. The process of tube formation was monitored every 3 h and the pictures were taken by using a Box-Type Fluorescence Imaging Device (OLYMPUS). The numbers of the tubes and branches in each well were counted.

### Quantitative real-time PCR

Total RNA from tumor tissues was isolated by a MicroElute Total RNA kit (Omega), followed by cDNA synthesis with a RevertAid First Strand cDNA Synthesis Kit (Thermo Scientific). The levels of related mRNA were determined by using a high-throughput fluorescence quantitative PCR meter (LightCycler480 II) (Roche). The primers (Table 1) were specifically designed to avoid nonspecific amplification by one half hybridizing to the 3′ end of one exon and the other half hybridizing to the 5′ end of the adjacent exon. Beta-actin was used as a reference gene to calculate the differences in gene expression (fold change).

**Table 1:** Primers used for qPCR analysis

Gene	Primer	Sequence (5′-3′)
*β-actin*	Forward Reverse	ATCGTGCGTGACATCAAAGA ACAGGATTCCATACCCAAGAAG
*Cxcl9*	Forward Reverse	AGTGTGGAGTTCGAGGAACC GAGTCCGGATCTAGGCAGG
*Ifnγ*	Forward Reverse	CCAAGTTTGAGGTCAACAACCC GGGACAATCTCTTCCCCACC
*Tnfα*	Forward Reverse	CCGATGGGTTGTACCTTGTC CGGACTCCGCAAAGTCTAAG
*Ang1*	Forward Reverse	ATGGAAAATTATACTCAGTGGCTGC ATTTAGTACCTGGGTCTCAACATC
*Tsp1*	Forward Reverse	TGTCACTGCCAGAACTCGGTTA GGAGACCAGCCATCGTCAG
*Timp1*	Forward Reverse	GAGACACACCAGAGCAGATACC GCTGGTATAAGGTGGTCTCGT

### Flow cytometry analysis

Mice bearing tumors were intracardially perfused with PBS. Tumor tissues were isolated and single cell suspensions were prepared by using the digesting DMEM medium containing collagenase type 1A (1500 U/ml), hyaluronidase (1000 U/ml) and DNase (20 U/ml). Rat anti-mouse CD16/CD32 monoclonal antibody was added into the single-cell suspensions before other antibody staining. After staining, cells were washed with cold flow buffer (1% BSA, 0.1% NaN_3_ in PBS), and 7AAD reagent (eBioscience) was added (5 ul/tube) prior to running the flow analysis. For intracellular cytokine staining, 2 million cells were cultured in a 12-well plate for 4 h with Brefeldin A (10 μg/ml, eBiosciences), and then stained with the surface antibody mixture. After washed, intracellular staining was performed according to the manufacturer’s instructions of a Fixation/Permeabilization Solution Kit (BD Bioscience). Flow cytometry data were acquired on a Gallios flow cytometer (Beckman) and analyzed with a Kaluza software (version 1.3). The following fluorescence-labeled or isotype-matched anti-mouse antibodies were used: Ly-6G-FITC, CD8-FITC, CD4-PE, NK1.1-APC-Cy7, Ly-6C-PE, Gr1-APC-Cy7, IFNγ-PE-Cy7, and IFNγ-APC (BD Biosciences); F4/80-APC, CD45-BV421, and CD11b-BV510 (BioLegend).

### In vivo IFNγ neutralization

When LAP0297 lung tumors reached 4–5 mm in diameter, mice bearing tumors were randomly divided into four groups, which were treated with an isotype-matched control rat IgG1 (clone HRPN, Bio-X Cell), 1 mg/kg LNT, 250 μg anti-IFNγ antibody (clone XMG1.2, Bio-X Cell), or LNT plus an anti-IFNγ antibody. LNT was administered into mice via *i.p.* injection every 3 days, while rat IgG1 or anti-IFNγ antibody were given every 3 days. The treatment duration was 10 days.

### Enzyme-linked immunosorbent assay (ELISA)

The concentrations of angiostatic factors IFNγ, TNFα, CXCL9, Ang1, TSP1, and TIMP1 in the tumor tissue lysate were measured according to the manufacturer’s protocols by using the following mouse ELISA Kits: IFNγ (Cat#: DKW12–2000-096, Dakewe, Shanghai, China), TNFα (Cat#: DKW12–2720-096, Dakewe, Shanghai, China), CXCL9 (Cat#: 70-EK21432/2, MultiSciences, Hangzhou, China), Ang1 (Cat#: DL-angpt1-mu-96 t), TIMP1 (Cat#: DL-timp1-mu-96 t), and TSP1 (Cat#: DL-thbs1-mu-96 t) from DLDEVELOP (Wuxi, China). Although different kits have different measure protocols, the major procedure is similar. Here, we used IFNγ measurement as an example of the ELISA method. Briefly, IFNγ standards, blank control and tested samples (100 μl/well) were added into 96-well plates. Then Biotinylated antibody (50 μl/well) was added to each well and incubated at 37 °C for 90 mins. After washing away unbound Biotinylated antibody, Streptavidin-HRP (100 μl/well) was added to each well and incubated for 30 mins. After washing, TMB (100 μl/well) was added and incubated at 37 °C for 10 mins in the dark. The reaction was discontinued by the Stop solution and the optical density (OD) values were determined at the wavelength of 450 nm by a microplate reader. The concentrations of angiostatic factors were calculated using the standard curve’s regression equation derived from standard absorbance values.

### Statistical analysis

Statistical analyses were performed using a Prism statistical software (version 6, GraphPad Software, Inc.). Unpaired 2-tailed Student’s *t* tests were used to determine the statistical differences between two groups. One-way analysis of variance (ANOVA) was used to assess the differences when more than two groups were compared. Data were presented as mean ± standard error of the mean (SEM). The results were considered as statistically significant at *P* < 0.05 (*). *P* values lower than 0.01 or 0.001 were indicated as “**” or “***”, respectively.

## Results

### LNT treatments exhibit better tumor growth inhibition at a relatively low dosage

**Fig. 1:**
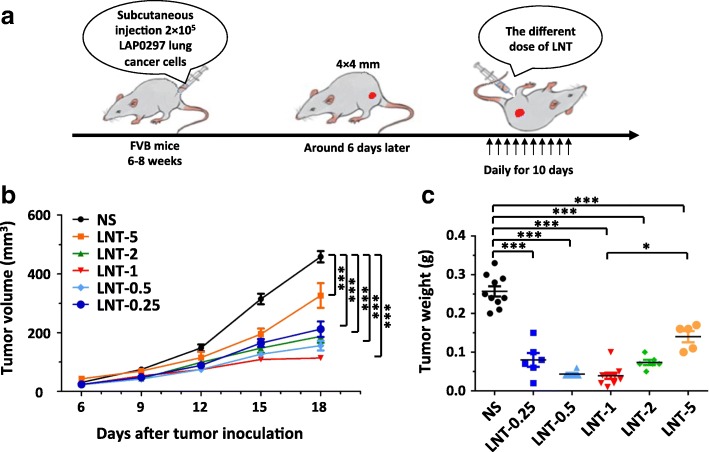
Lentinan (LNT) exhibits better antitumor effects at a relatively lower dose compared with a higher dose. **a** Experimental design: FVB mice were subcutaneously (*s.c.*) inoculated with 2 × 10^5^ LAP0297 lung cancer cells on the right flank. When tumors reached 4 × 4 mm in diameter, mice were randomly divided into 6 groups and received *i.p.* injection of different doses of LNT (0.25 mg/kg, 0.5 mg/kg, 1.0 mg/kg, 2.0 mg/kg, and 5.0 mg/kg) daily for 10 days. **b** LAP0297 lung tumor-bearing mice received different doses of LNT treatments. **c** Tumor weight was measured at the end of the treatments. NS: control group treated with saline. All data in this report are presented as means ± SEM. * *p* < 0.05, ** *p* < 0,01, *** *p* < 0.001

Tumor immune evasion and tumor angiogenesis are two hallmarks of cancer [2, 22, 25]. LNT has been shown to suppress the growth of several types of cancer and the effects have been largely attributed to immune stimulation [13, 14, 16, 18, 26, 27]. Whether LNT intervention influences tumor angiogenesis remains unclear. To determine the impact of LNT on tumor angiogenesis, we initially evaluated the dose effects of LNT treatments on LAP0297 lung carcinoma. We treated tumor-bearing mice with 0.25, 0.5, 1.0, 2.0, or 5.0 mg/kg LNT for 10 days (Fig. 1a). All tested dosages of LNT treatments inhibited LAP0297 lung tumor growth, however, relatively lower doses exhibited better tumor control than higher doses, suggesting that LNT treatment inhibited tumor growth in an inverted U-shaped dose-response manner. Our data showed that the 1.0 mg/kg LNT treatments exhibited better antitumor effects, compared to the 5.0 mg/kg LNT treatments (Fig. 1b-c). Based on these results, we chose 1.0 mg/kg as the standard LNT treatment dose for LAP0297 lung tumors in the rest of this study. This unique dose effect of LNT was also observed in the CT26 colorectal cancer model, where 1.0 mg/kg LNT treatments inhibited tumor growth, but 4.0 mg/kg LNT treatments did not affect tumor growth, again showing that a lower dose of LNT treatment has better antitumor effects (Additional file 1: Figure S1). Taken together, these data show that appropriately lower doses of LNT treatments exhibit better antitumor effects, compared to those of higher doses.

### LNT treatments reduce tumor vascular function in lung cancer

**Fig. 2:**
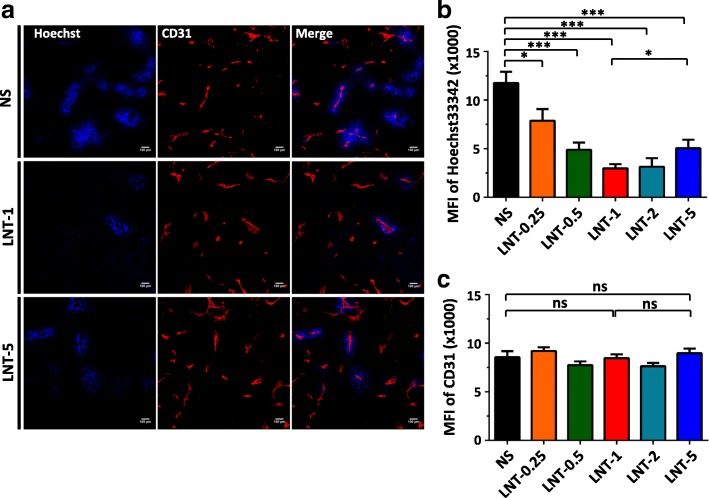
LNT treatments reduce tumor vessel function in an inversed U-shaped dose-response manner. LAP0297 tumor bearing mice were treated with different doses of LNT for 10 days as described in Fig. 1. Tumor tissues were sectioned and stained with an anti-CD31 antibody. **a** Representative figures showing tumor vessel density and vascular function in NS control and LNT (1 and 5 mg/kg) groups. **b** The mean fluorescence intensity (MFI) of CD31 (Red) and Hoechst 33342 (Blue) stained areas was quantified. CD31 is an endothelial cell marker. The intensity of Hoechst 33342 perfusion reflects the function of tumor vessels. ns: no significantly different, * *p* < 0.05, *** *p* < 0.001

**Fig. 3:**
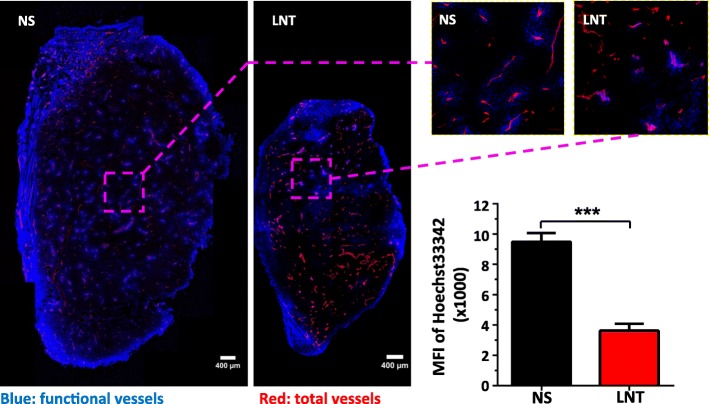
The whole images of tumor tissue cross-sections show reduced tumor vascular function after LNT treatments in LAP0297 lung cancer. LAP0297 tumor bearing mice received saline or LNT treatments (1.0 mg/kg) for 10 days as described in Fig. 1. Tumor tissues were sectioned and stained with an anti-CD31 antibody. The proportions of endothelial cell area (CD31, Red) and Hoechst 33342 (Blue) stained area over total tumor tissue area were quantified. CD31 is an endothelial cell marker. The intensity of Hoechst 33342 perfusion reflects the function of tumor vessels. *** *p* < 0.001

We next investigated the effects of LNT treatments on tumor angiogenesis. Vessel density is a common parameter to evaluate the effects of an intervention on the tumor vasculature [22]. After 10 days of LNT treatments in the LAP0297 lung cancer model, vessel density in all of LNT-treated groups was comparable to the control group, even though tumors were smaller in LNT-treated groups when compared to the control group (Figs. 1 and 2). We then analyzed other parameters to get more information about the tumor vasculature. Interestingly, we found that LNT treatments suppressed tumor vascular function in an inverted U-shaped dose-response manner (Fig. 2). A relatively lower treatment dose of LNT (1.0 mg/kg) exhibited stronger antiangiogenic effects, compared to a relatively higher dose (5.0 mg/kg) (Fig. 2). Given that the tumor microenvironment is highly heterogeneous and tumor vessels are unevenly distributed [28, 29], we next looked at the whole images of the entire cross-sections of tumor tissues and analyzed the overall tumor vascular function. Again, the data showed that LNT treatments (1.0 mg/kg) decreased tumor vascular function (Fig. 3). To evaluate whether LNT treatments affect blood vessels in normal tissues, we examined blood vessels in colon tissues and found that LNT treatments didn’t alter their vessel density or function (Additional file 1: Figure S2). Consistent to the reduction in tumor vascular function, the necrotic area in the LNT-treated group was increased compared to the control group (Additional file 1: Figure S3). Taken together, our data suggest that LNT treatments suppress tumor angiogenesis in the lung cancer model.

### LNT treatments suppress tumor vascular function in colorectal cancer

**Fig. 4:**
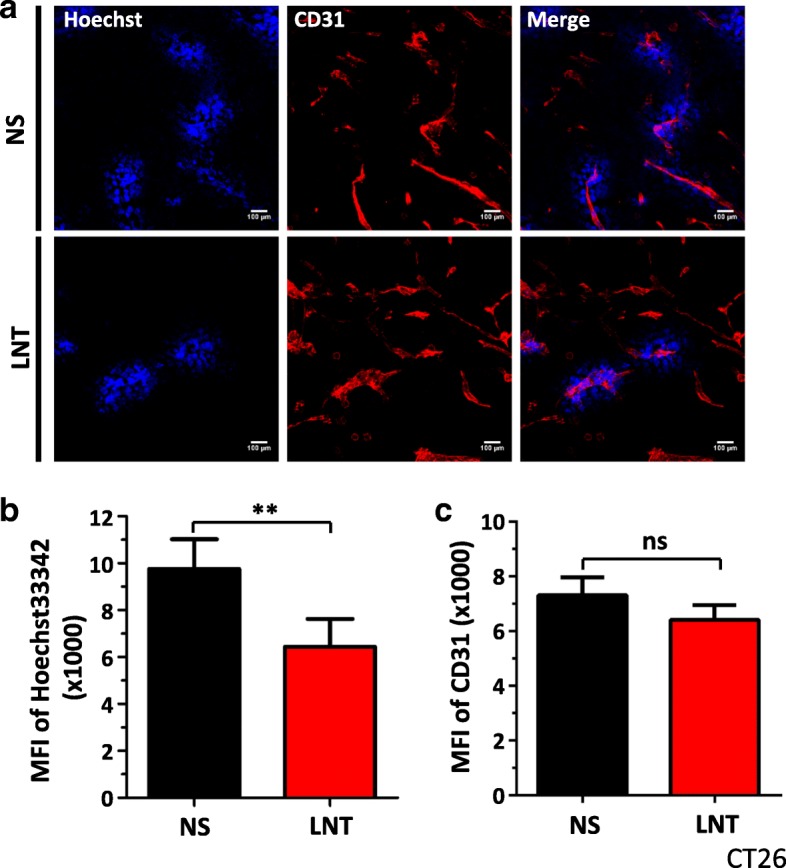
LNT treatments significantly reduce tumor vascular function in CT26 colorectal carcinoma. CT26 colorectal carcinoma cells (4 × 10^5^) were *s.c.* injected on the right flank of BALB/c mice. When tumors reached 4 × 4 mm in diameter, mice were randomly divided into 2 groups and received *i.p.* injection of saline or 1.0 mg/kg of LNT for 10 days. Tumor vascular parameters were determined as described in Fig. 2. **a** Representative figures showed tumor vessel density and vascular function. **b** The quantifications of MFI of Hoechst33342 perfused tumor areas in saline (NS) or LNT treated tumors. **c** The MFI of tumor vessel density in saline or LNT treated tumors. ** *p* < 0.01

To determine whether the antiangiogenic effects of LNT treatments are tumor-type dependent, we treated mice bearing CT26 colorectal cancer with LNT. Consistent with the effects seen in the LAP0297 lung cancer model, 10 days of LNT treatments (1.0 mg/kg) inhibited CT26 tumor growth (Additional file 1: Figure S1). We then analyzed the effects of LNT treatments on tumor angiogenesis. LNT treatments (1.0 mg/kg) significantly decreased the Ho33342 perfused tumor area, but did not change the density of tumor blood vessels (Fig. 4). The data show that LTN treatments inhibit colorectal tumor vascular function, indicating that LNT can be used as an antiangiogenic agent for colorectal cancer treatment.

### Long-term LNT treatments induce the regression of lung cancer

**Fig. 5:**
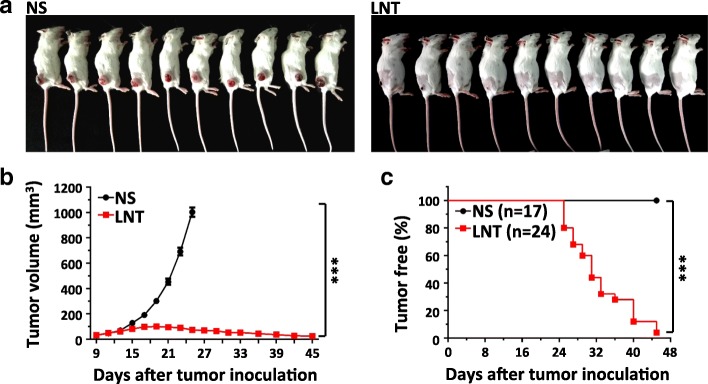
Long-term LNT treatments induce regression of LAP0297 lung cancer. LAP0297 tumor bearing mice were prepared as described in Fig. 1. When tumors reached 4 × 4 mm in diameter, mice were randomly divided into 2 groups and received *i.p.* injection of saline or LNT (1.0 mg/kg) for 1 month. **a** Representative photographs of LAP0297 lung cancer-bearing mice in saline and LNT-treated group were taken at the end of the experiment. **b** The growth curves of LAP0297 lung cancer upon long-term therapy of saline or LNT. **c** The percentage of tumor free mice on day 45 after tumor inoculation (NS group, *n* = 17 mice, LNT-treated group, *n* = 24 mice). *** *p* < 0.001

Antiangiogenic therapy is widely used in the clinic and shows modest antitumor efficacy in several cancer types, such as NSCLC and colorectal cancer. One crucial challenge for antiangiogenic therapy is the emergence of rapid drug resistance [1, 6, 29]. To determine the long-term antiangiogenic effect of LNT treatment, we continuously treated mice bearing LAP0297 lung cancer with LNT (1.0 mg/kg) for 1 month. During the LNT treatment course, the size of LAP0297 lung cancers persistently reduced and about 60% of tumors were completely regressed at the end of the treatment course (Fig. 5). We kept monitoring the tumor growth when LNT treatment was terminated, and found that there was no observed LAP0297 lung tumor regrowth 2 weeks post the LNT treatment course, and the percentage of tumor free mice reached 95% (Fig. 5). These results suggest that long-lasting LNT treatments exhibit persistent antitumor effects in the LAP0297 lung cancer model.

### LNT treatments upregulate the expression of angiostatic factors

To understand the mechanism of LNT inhibition against tumor angiogenesis, we performed endothelial cell tube formation assays, and found that LNT treatments did not directly affect endothelial cell tube formation (Additional file 1: Figure S4). We then analyzed the transcription of angiogenesis-related genes, and found that angiostatic genes, including *interferon γ (Ifnγ)*, *tumor necrosis factor α (Tnfα)*, *chemokine (C-X-C motif) ligand 9* (*Cxcl9*), *Timp1*, and *Thrombospondin 1* (*Tsp1)*, were up-regulated in LNT-treated tumors, compared to control tumors (Additional file 1: Figure S5). Moreover, we conducted ELISA to determine the protein levels of the angiostatic factors in the tumor microenvironment. The results demonstrated that LNT treatments (1 mg/kg) elevated the protein levels of IFNγ, TNFα, CXCL9, and TIMP1 (Additional file 1: Figure S6). These data collectively suggest that LNT treatments induce the expression of angiostatic factors.

### LNT treatments increase IFNγ production in T cells and myeloid cells

**Fig. 6:**
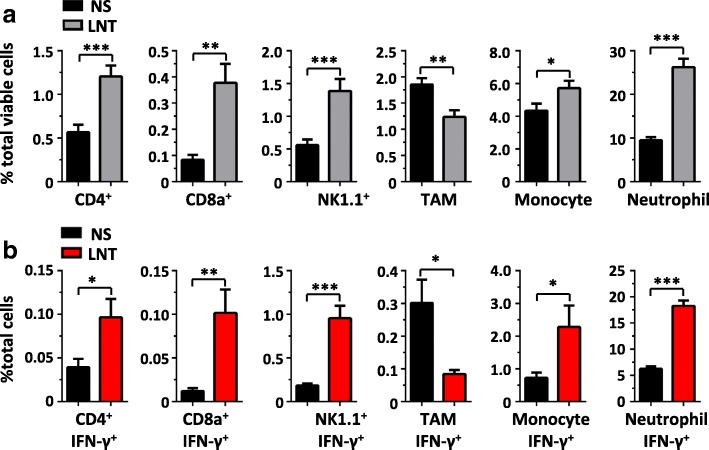
LNT treatments promote tumor infiltration of IFNγ-expressing T cells and myeloid cells. LAP0297 tumor bearing mice were prepared and then treated with saline or 1.0 mg/kg LNT for 10 days as described in Fig. 1. Tumor tissues were isolated and single cell suspensions were prepared for flow analysis. **a** Tumor infiltration of T cells and myeloid cells. **b** The proportions of IFNγ-expressing T cells and myeloid cells in LAP0297 tumor tissues upon saline or LNT treatments. * *p* < 0.05, ** *p* < 0,01, *** *p* < 0.001

Since IFNγ is often secreted by immune cell populations upon stimulation, we next analyzed the effects of LNT treatments on tumor-infiltrating immune cells. Flow cytometric analysis showed that LNT treatments facilitated tumor infiltration of CD4^+^, CD8^+^, NK1.1^+^ cells, monocytes, and neutrophils, while reducing tumor-associated macrophages (TAMs) (Fig. 6a). Intracellular staining further showed that LNT treatments increased the fractions of IFNγ-expressing T cells and NK cells, as well as IFNγ-expressing monocytes and neutrophils, but not TAMs (Fig. 6b). The data suggest that LNT treatments promote the accumulation of IFNγ-expressing T cells and myeloid cells.

### IFNγ is required for LNT treatment to reduce tumor vascular function

**Fig. 7:**
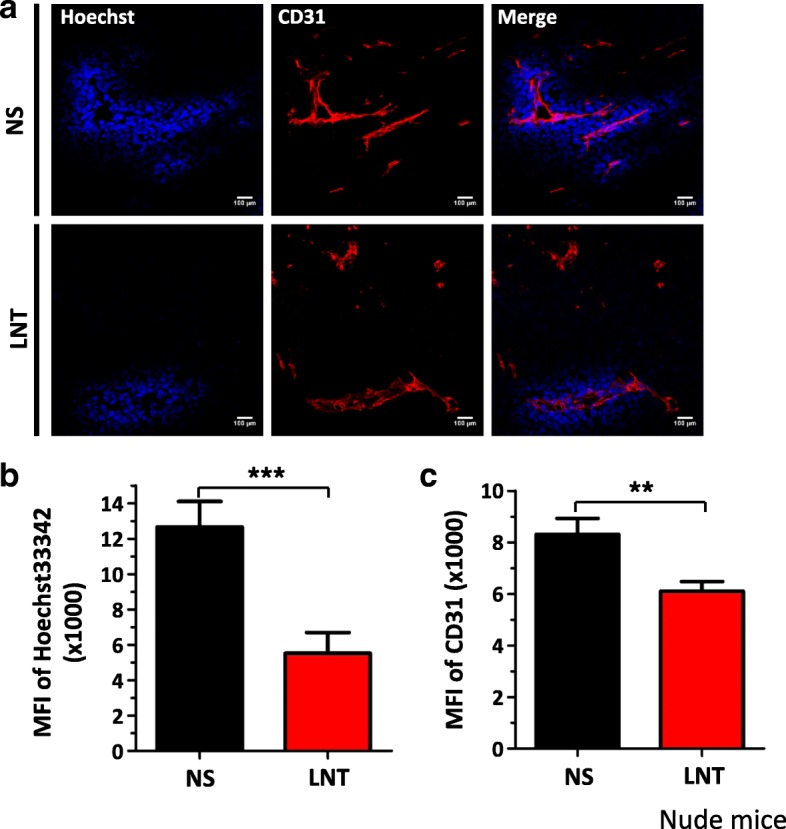
LNT treatments reduce tumor vascular function in LAP0297 lung cancer in nude mice. Nude mice were *s.c.* inoculated with 2 × 10^5^ LAP0297 lung cancer cells on the right flank. When tumors reached 4 × 4 mm in diameter, mice were randomly divided into 2 groups and received i.p. injection of saline or 1.0 mg/kg of LNT for 10 days. Tumor vascular parameters were determined as described in Fig. 2. **a** Representative figures showing tumor vessel density and function. The MFI of Hoechst33342 perfused tumor areas **b** and the MFI of tumor vessel density **c** in saline or LNT treated tumors. ** *p* < 0.01, *** *p* < 0.001

Previous studies have suggested that IFNγ-expressing T cells could suppress tumor angiogenesis [8, 12]. Together with the reports that LNT is commonly used as an immune modulator, and that T cells are often major antitumor immune effector cells [13, 14, 18, 28], we thus tested whether LNT exerts its antiangiogenic effects through T cells. We inoculated LAP0297 lung cancer cells in nude mice and then treated mice with 1.0 mg/kg LNT for 10 days as did in immune competent mice (Fig. 1). We found that LNT treatments inhibited the growth of LAP0297 lung cancer in nude mice (Additional file 1: Figure S7), but the degree of inhibition was less than that in immune competent mice (Fig. 1), indicating that T cells contribute to the inhibition of tumor growth by LNT treatments. This is consistent with the immunopotentiation of LNT. We also analyzed tumor vessels, and found that LNT treatments suppressed tumor vascular function in nude mice (Fig. 7), and that the degree of suppression was comparable to those of immune competent mice (Fig. 2). LNT treatments also slightly reduced tumor vessel density (Fig. 7), which was not observed in immune competent mice (Fig. 2). These data suggest that LNT treatments inhibit tumor angiogenesis in a T cell-independent manner.

**Fig. 8:**
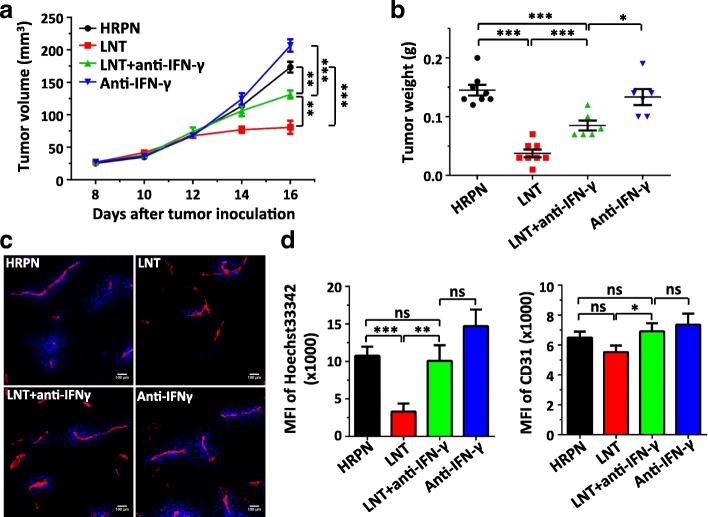
Anti-IFN-γ antibody treatments reverse the effects of LNT treatments on tumor vessels in LAP0297 lung cancer. LAP0297 tumor bearing mice were prepared as described in Fig. 1. When tumors reached 4 × 4 mm in diameter, mice were randomly divided into 4 groups and received *i.p.* injection of HRPN, LNT (1.0 mg/kg), anti-IFN-γ antibody (250 μg/mouse) or LNT plus anti-IFN-γ antibody for 10 days. Tumor vascular parameters were determined as described in Fig. 2. **a** and **b** The growth curves and weight of LAP0297 tumors treated with HRPN, LNT, anti-IFN-γ antibody, or the combination of LNT and anti-IFN-γ antibody. **c** Representative figures showing tumor vascular function and vessel density. **d** The quantifications of Hoechst33342 perfused tumor areas and tumor vessel density. ns: no significantly different, * *p* < 0.05, ** *p* < 0.01, *** *p* < 0.001

A recent study demonstrated that elevated levels of IFNγ arrested tumor blood flow without affecting tumor endothelial cell proliferation and apoptosis [10]. To further explore the relationship between IFNγ and LNT treatment, we tested whether IFNγ is responsible for the observed LNT-mediated suppression of vascular function. In vivo neutralization of IFNγ partially reversed the inhibition of tumor growth by LNT treatments compared with the control group, while IFNγ neutralization alone had little effect on tumor growth (Fig. 8a-b). Consistent with previous data, LNT treatments alone reduced tumor vascular function, and IFNγ neutralization negated the effect of LNT treatments on tumor vascular function, indicating that IFNγ mediates the impact of LNT treatments on tumor vascular function (Fig. 8). Taken together, these data suggest that LNT treatments suppress tumor angiogenesis via IFNγ and in a T cell-independent manner, which is correlated with an increase in IFNγ-expressing myeloid cells.

## Discussion

Tumor initiation and progression relies on the formation of new blood vessels. Thus, the disruption of tumor blood vessels has the potential to inhibit tumor growth. However, the clinical benefits of antiangiogenic therapies are often in the order of weeks and rapidly developed drug resistance limits its long-term application [1, 6, 29]. In this study, we found that both of T cells and IFNγ production contribute to tumor growth inhibition induced by LNT treatments, whereas IFNγ, but not T cells, is required for the LNT-mediated antiangiogenic effect. Moreover, prolonged LNT treatments persistently suppressed tumor angiogenesis and reduced tumor volume. These results suggest that LNT could be served as a new antiangiogenic agent and may be suitable for long-term intervention.

LNT is a polysaccharide from the fruit body of *L. edodes* and has been used previously as a biological response modifier [14, 15, 30]. In particular, LNT has been approved as an adjuvant for the treatment of gastric cancer and brought clinical benefits to cancer patients [16, 31, 32]. LNT has demonstrated its antitumor effects in both primary and transplanted tumor models with negligible side effects [17, 33, 34]. Our data further suggest that LNT treatments decrease tumor vascular function, but do not affect vascular function in normal tissues. Such difference effects could be due to the fact that LNT treatments suppress tumor angiogenesis via increasing IFNγ production, which is associated with the accumulation of IFNγ-expressing neutrophils. Normal tissues usually contain few neutrophils and will show minimal effects by LNT treatments. In addition, LNT could alleviate side toxicities of chemotherapeutic agents and potentially improve their efficacy [26, 35]. The safety profiles of LNT as well as its ability to overcome the side effects of chemotherapy is superior to currently used traditional antiangiogenic drugs, providing new rationales for developing LNT as an antiangiogenic agent.

Blood vessel formation is tightly regulated by pro- and anti-angiogenic factors. The relentless production of pro-angiogenic factors promotes tumor vessel formation [3, 29]. Thus antiangiogenic therapy is usually designed to suppress pro-angiogenic factors and inhibit tumor progression [1, 3]. Currently, antiangiogenic agents are used in the treatments of various types of solid cancers, such as NSCLC and colorectal cancer [1, 3]. Therefore, we chose LAP0297 lung carcinoma and CT26 colorectal cancer as tumor models to evaluate the effects of LNT treatments on tumor angiogenesis. Although antiangiogenic therapy improves chemotherapy in several cancer types, the clinical benefits are marginal. One of the critical reasons is the development of drug resistance. Some tumors are intrinsic resistance to antiangiogenic therapy [3, 29]. Some tumors respond to antiangiogenic therapy at the beginning, but develop drug resistance later on [1]. With antiangiogenic treatments being susceptible to the development of drug resistance, it is significant that we treated LAP0297 lung carcinoma with LNT for 1 month and did not observed drug resistance. Gene transcription and protein expression data showed that LNT treatments upregulated the levels of IFNγ, TNFα, CXCL9, and TIMP1. Among them, TIMP1 is an intrinsic angiostatic factor, while IFNγ, TNFα, and CXCL9 are immune effector molecules with potent angiostatic activities. Whether or not elevated endogenous angiostatic factors will be less likely to cause drug resistance is not known, but the phenomenon is very interesting and worth further investigations.

Therapeutic antitumor drugs often suppress tumor growth in a dose-dependent manner. In general, higher dosages show better antitumor effect than that of lower dosages. The optimal therapeutic dosage is usually determined by the efficacy and toxicity of the drug. Interestingly, LNT inhibited tumor growth in an inversed U-shaped dose-response manner. Appropriately lower dose of LNT (such as 1.0 mg/kg) showed better antitumor efficacy than that of higher dose (such as 5.0 mg/kg). The exact underlying mechanism of this action is unknown. It could be due to the anti-angiogenic effects of LNT treatments. Our data showed that a relatively lower dose of LNT treatments (such as 1.0 mg/kg) more potently reduced tumor vascular function than that of a relatively higher dose (such as 5.0 mg/kg). Furthermore, our study suggests that LNT treatments (1.0 mg/kg) inhibit tumor vascular function via IFNγ production and in a T cell-independent manner. In addition, LNT treatments (1.0 mg/kg) up-regulated IFNγ production in several tumor-infiltrating myeloid cell populations. It is possible that the optimal treatment dose of LNT could be different for different populations of immune cells. The optimal treatment dose of LNT on a specific population of immune cells with respect to their quantity and function could be also different. Indeed, the 1.0 mg/kg treatment of LNT, but not the 5.0 mg/kg treatment of LNT, increased tumor infiltration of neutrophils (Additional file 1: Figure S8), which is the most abundant tumor-infiltrating myeloid cell population in LAP0297 lung carcinoma (Fig. 6a). Therefore, the optimal antitumor effects of LNT treatments seem to depend on the balance of vessel modulation and immune stimulation upon LNT treatments.

Since LNT treatment inhibits tumor growth in an inversed U-shaped dose-response manner, however, the lower or higher dose of LNT is relative and could be difficult to determine. This is an even bigger challenge in the clinic because different patients and different cancer types may have different sensitivities to LNT treatments. Our previous work suggests that monitoring vascular function could be used to predict the efficacy of immune checkpoint therapy [36]. In this study, our findings showed that a relatively lower dose of LNT treatments was more powerful than a higher dose in decreasing tumor vessel perfusion. Given that radiological methods have been developed to noninvasively measure vessel perfusion during anti-angiogenic therapy in the clinic [37, 38], it is conceivable that vessel perfusion monitoring could be adapted to determine the optimal dosage of LNT treatment.

LNT has been applied in some kinds of cancer treatments. Its antitumor effects are usually considered to be the result of its immune stimulation. Several recent studies suggested that LNT could induce tumor cell apoptosis, showing directly antitumor effect [30, 39]. By using lung and colorectal cancer models, we showed that LNT treatments inhibited tumor angiogenesis via increased IFNγ production in a T cell-independent manner. Taken together, LNT could affect tumor growth via multiple different mechanisms, including the modulation of immune system, the induction of tumor cell apoptosis [13, 14, 16, 18, 30, 39], and the suppression of tumor angiogenesis.

## Conclusions

LNT treatments reduced tumor vascular perfusion and inhibited tumor growth in an inverted U-shaped dose-response manner. Furthermore, long-term LNT treatments continuously suppressed tumor angiogenesis and exhibited antitumor effects in LAP0297 lung tumor model. Mechanically, LNT decreased tumor vascular function by increasing IFNγ production and in a T cell-independent manner, which was associated with the accumulation of tumor-infiltrating myeloid cells. Thus, this study suggests that LNT could be served as a new antiangiogenic agent for long-term cancer treatments.

## Additional file

Additional file 1: **Methods:** CT26 colorectal carcinoma cells (4x10^5^) or LAP0297 lung carcinoma cells (2x10^5^) were *s.c.* injected on the right flank of BALB/c or FVB mice, respectively. When tumors reached 4×4 mm in diameter, mice were randomly divided into 2 groups and received *i.p.* injection of saline (NS) or 1.0 mg/kg of LNT daily for 10 days. At the end of the treatments, tumor tissues were isolated, sectioned, and stained by an anti-CD31 antibody (endothelial cells). The proportions of endothelial cell area (CD31, Red) and Hoechst 33342 (Blue) perfused area over total tumor tissue area were quantified. SYTOX Green (Green) was used to label total cells. All data in the Additional file 1 are presented as means ± SEM. * p<0.05, ** p<0.01, *** p<0.001, and “ns” designates no significant difference. **Fig. S1** LNT treatments inhibit tumor growth of CT26 colorectal carcinoma. **a** The experimental design of CT26 colorectal tumor model. **b** The growth curves and weight of CT26 colon tumors treated with saline or LNT. **Fig. S2** LNT treatments do not affect the vasculature in normal colon tissues in LAP0297 tumor-bearing mice. **Fig. S3** LNT treatments increase necrotic area. LAP0297 tumor tissues were sectioned and SYTOX Green (Green) was used to label tumor tissue cells. Necrotic cells were relatively small, dense, and round. **Fig. S4** LNT treatments do not affect endothelial cell tube formation in vitro. HUVECs were seeded on the top of growth factor reduced matrigel in a 24-well plate, and then incubated with different concentrations of LNT. The process of tube formation was monitored every 3 hrs and pictures were taken at 12 hrs after incubation with LNT. The tubes and branches in each well were counted. **Fig. S5** LNT treatments up-regulate the transcription of angiostatic factors, such as *Ifnγ*, *Tnfα*, *Cxcl9*, *Ang1*, *Timp1*, and *Tsp1*, in LAP0297 tumor tissues. **Fig. S6** LNT treatments increase the expression of angiostatic factors, such as IFNγ, TNFα, CXCL9, Ang1, TIMP1, and TSP1, in LAP0297 tumor tissues. **Fig. S7** LNT treatments inhibit tumor growth of LAP0297 lung cancer in nude mice. Nude mice were *s.c. *injected with 2×10^5^ LAP0297 lung cancer cells on the right flank and treatments were performed as described in the above methods. Tumor sizes were measured every three days. **Fig. S8** Relatively lower dose of LNT treatments (1 mg/kg), but not higher dosage (5 mg/kg), promotes the tumoral accumulation of neutrophils. LAP0297 tumor tissues were isolated and single cell suspensions were prepared. The tumor infiltration of leukocytes (CD45), neutrophils (Gr1^hi^), and TAMs were determined by flow analysis. (ZIP 7212 kb)

## Abbreviations

CXCL9: Chemokine (C-X-C motif) ligand 9
FGF: Fibroblast growth factor
IFNγ: Interferon gamma
LNT: Lentinan
NSCLC: Non-small cell lung cancer
PDGF: Platelet-derived growth factor
PlGF: Placental growth factor
TIMP1: TIMP metallopeptidase inhibitor 1
TKI: Multiple targeted kinase inhibitors
TNFα: Tumor necrosis factor alpha
TSP1: Thrombospondin 1
VEGF: Vascular endothelial growth factor
