# Correction to: Lentinan inhibits tumor angiogenesis via interferon γ and in a T cell independent manner

**DOI:** 10.1186/s13046-021-02170-8

**Published:** 2021-11-18

**Authors:** Shengming Deng, Guoxi Zhang, Jiajie Kuai, Peng Fan, Xuexiang Wang, Pei Zhou, Dan Yang, Xichen Zheng, Xiaomei Liu, Qunli Wu, Yuhui Huang

**Affiliations:** 1 Cyrus Tang Hematology Center, Collaborative Innovation Center of Hematology, State Key Laboratory of Radiation Medicine and Prevention, Soochow University, 199 Ren-Ai Road, Suzhou, 215123 Jiangsu China; 2 Nanjing Luye Pharmaceutical Co., Ltd, Nanjing, 210061 Jiangsu China; 3 Department of Traditional Chinese Medicine, Peking Union Medical College Hospital, Peking Union Medical College and Chinese Academy of Medical Sciences, No. 1 Shuaifuyuan, Dongcheng District, Beijing, 100730 China

**Correction to: J Exp Clin Cancer Res 37, 260 (2018)**

**https://doi.org/10.1186/s13046-018-0932-y**

Following publication of the original article [1], the authors identified minor errors in Fig. 5; specifically, in Fig. 5c, the label ‘Tumor free (%)’ was used. The correct label is ‘Tumor-bearing mice (%)’. The corrected figure is provided here; the figure caption has been corrected to reflect the same.

**Fig. 5:**
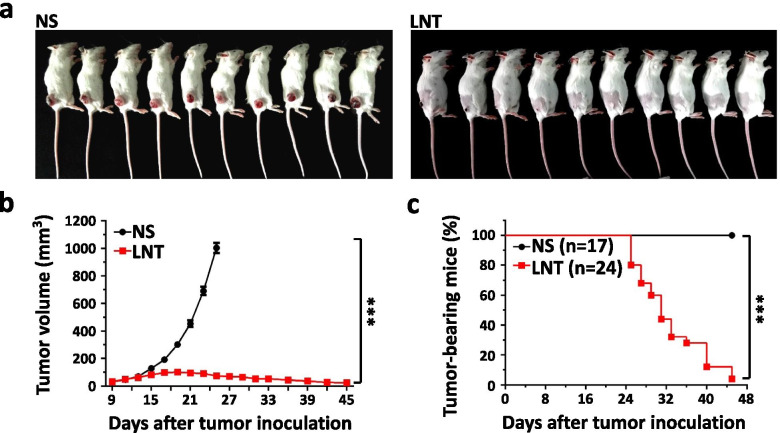
Long-term LNT treatments induce regression of LAP0297 lung cancer. LAP0297 lung cancer-bearing mice were prepared as described in Fig. 1. When tumors reached 4 × 4 mm in diameter, mice were randomly divided into 2 groups and received *i.p.* injection of saline or LNT (1.0 mg/kg) for 1 month. **a** Representative photographs of LAP0297 lung cancer-bearing mice in saline and LNT-treated group were taken at the end of the experiment. **b** The growth curves of LAP0297 lung cancer upon long-term therapy of saline or LNT. **c** The percentage of tumor-bearing mice on day 45 after tumor inoculation (NS group, *n* = 17 mice, LNT-treated group, *n* = 24 mice). *** *p* < 0.001

In addition, the Competing Interests section of the article has been corrected as follows:

**Competing interests**

Guoxi Zhang is an employee of Nanjing Luye Pharmaceutical Co., Ltd (Nanjing, China) and provided the main research subject (Lentinan) of the study. The other authors declare that they have no competing interests.

The correction does not have any effect on the results or conclusions of the paper. The original article has been corrected.
