# Supplementary figures and images for: Lentinan inhibits tumor angiogenesis via interferon γ and in a T cell independent manner
Source: J Exp Clin Cancer Res. 2018 Oct 29;37:260. doi: 10.1186/s13046-018-0932-y (PMC6206909; doi:10.1186/s13046-018-0932-y)

Fig. S1

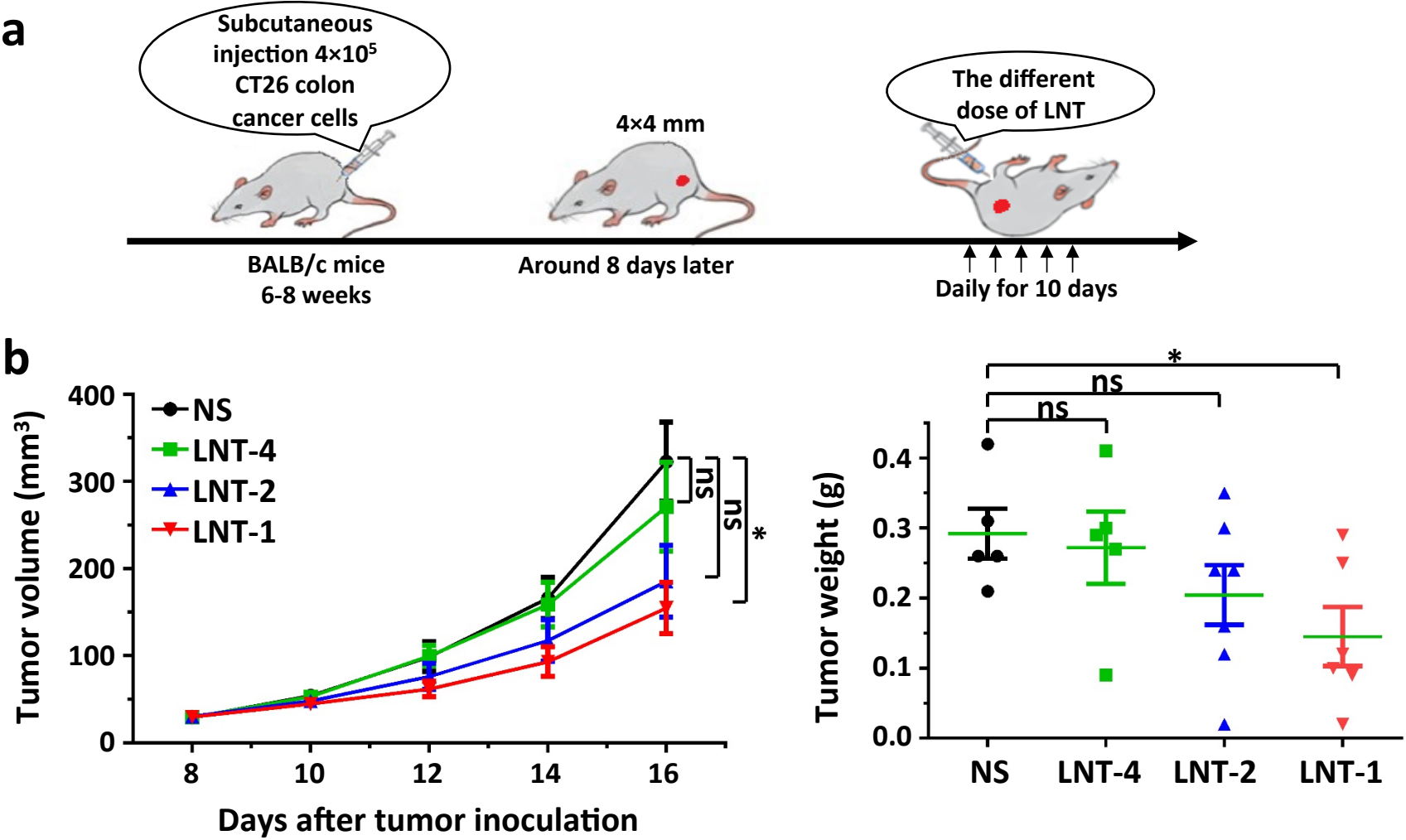

Supplement: Supplementary file 1 — Methods: CT26 colorectal carcinoma cells (4x10^5^) or LAP0297 lung carcinoma cells (2x10^5^) were s.c. injected on the right flank of BALB/c or FVB mice, respectively. When tumors reached 4×4 mm in diameter, mice were randomly divided into 2 groups and received i.p. injection of saline (NS) or 1.0 mg/kg of LNT daily for 10 days. At the end of the treatments, tumor tissues were isolated, sectioned, and stained by an anti-CD31 antibody (endothelial cells). The proportions of endothelial cell area (CD31, Red) and Hoechst 33342 (Blue) perfused area over total tumor tissue area were quantified. SYTOX Green (Green) was used to label total cells. All data in the Additional file 1 are presented as means ± SEM. * p<0.05, ** p<0.01, *** p<0.001, and “ns” designates no significant difference. Fig. S1 LNT treatments inhibit tumor growth of CT26 colorectal carcinoma. a The experimental design of CT26 colorectal tumor model. b The growth curves and weight of CT26 colon tumors treated with saline or LNT. Fig. S2 LNT treatments do not affect the vasculature in normal colon tissues in LAP0297 tumor-bearing mice. Fig. S3 LNT treatments increase necrotic area. LAP0297 tumor tissues were sectioned and SYTOX Green (Green) was used to label tumor tissue cells. Necrotic cells were relatively small, dense, and round. Fig. S4 LNT treatments do not affect endothelial cell tube formation in vitro. HUVECs were seeded on the top of growth factor reduced matrigel in a 24-well plate, and then incubated with different concentrations of LNT. The process of tube formation was monitored every 3 hrs and pictures were taken at 12 hrs after incubation with LNT. The tubes and branches in each well were counted. Fig. S5 LNT treatments up-regulate the transcription of angiostatic factors, such as Ifnγ, Tnfα, Cxcl9, Ang1, Timp1, and Tsp1, in LAP0297 tumor tissues. Fig. S6 LNT treatments increase the expression of angiostatic factors, such as IFNγ, TNFα, CXCL9, Ang1, TIMP1, and TSP1, in LAP0297 tumor tissues. Fi [file 13046_2018_932_MOESM1_ESM.zip › Figure S1.pdf]

**Fig. S2**

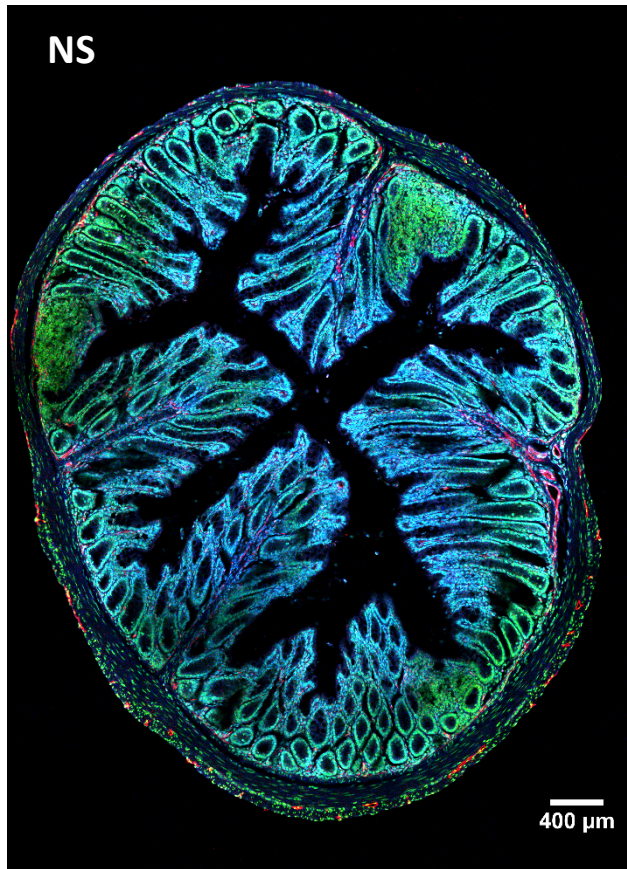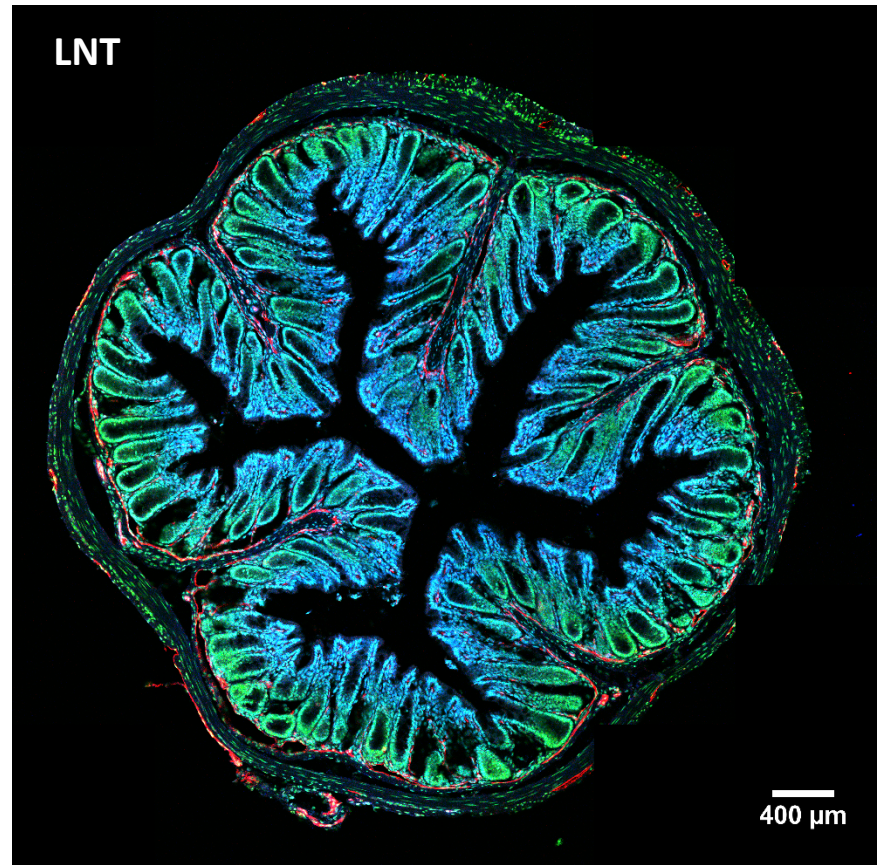

Supplement: Supplementary file 1 — Methods: CT26 colorectal carcinoma cells (4x10^5^) or LAP0297 lung carcinoma cells (2x10^5^) were s.c. injected on the right flank of BALB/c or FVB mice, respectively. When tumors reached 4×4 mm in diameter, mice were randomly divided into 2 groups and received i.p. injection of saline (NS) or 1.0 mg/kg of LNT daily for 10 days. At the end of the treatments, tumor tissues were isolated, sectioned, and stained by an anti-CD31 antibody (endothelial cells). The proportions of endothelial cell area (CD31, Red) and Hoechst 33342 (Blue) perfused area over total tumor tissue area were quantified. SYTOX Green (Green) was used to label total cells. All data in the Additional file 1 are presented as means ± SEM. * p<0.05, ** p<0.01, *** p<0.001, and “ns” designates no significant difference. Fig. S1 LNT treatments inhibit tumor growth of CT26 colorectal carcinoma. a The experimental design of CT26 colorectal tumor model. b The growth curves and weight of CT26 colon tumors treated with saline or LNT. Fig. S2 LNT treatments do not affect the vasculature in normal colon tissues in LAP0297 tumor-bearing mice. Fig. S3 LNT treatments increase necrotic area. LAP0297 tumor tissues were sectioned and SYTOX Green (Green) was used to label tumor tissue cells. Necrotic cells were relatively small, dense, and round. Fig. S4 LNT treatments do not affect endothelial cell tube formation in vitro. HUVECs were seeded on the top of growth factor reduced matrigel in a 24-well plate, and then incubated with different concentrations of LNT. The process of tube formation was monitored every 3 hrs and pictures were taken at 12 hrs after incubation with LNT. The tubes and branches in each well were counted. Fig. S5 LNT treatments up-regulate the transcription of angiostatic factors, such as Ifnγ, Tnfα, Cxcl9, Ang1, Timp1, and Tsp1, in LAP0297 tumor tissues. Fig. S6 LNT treatments increase the expression of angiostatic factors, such as IFNγ, TNFα, CXCL9, Ang1, TIMP1, and TSP1, in LAP0297 tumor tissues. Fi [file 13046_2018_932_MOESM1_ESM.zip › Figure S2.pdf]

**Fig. S3**

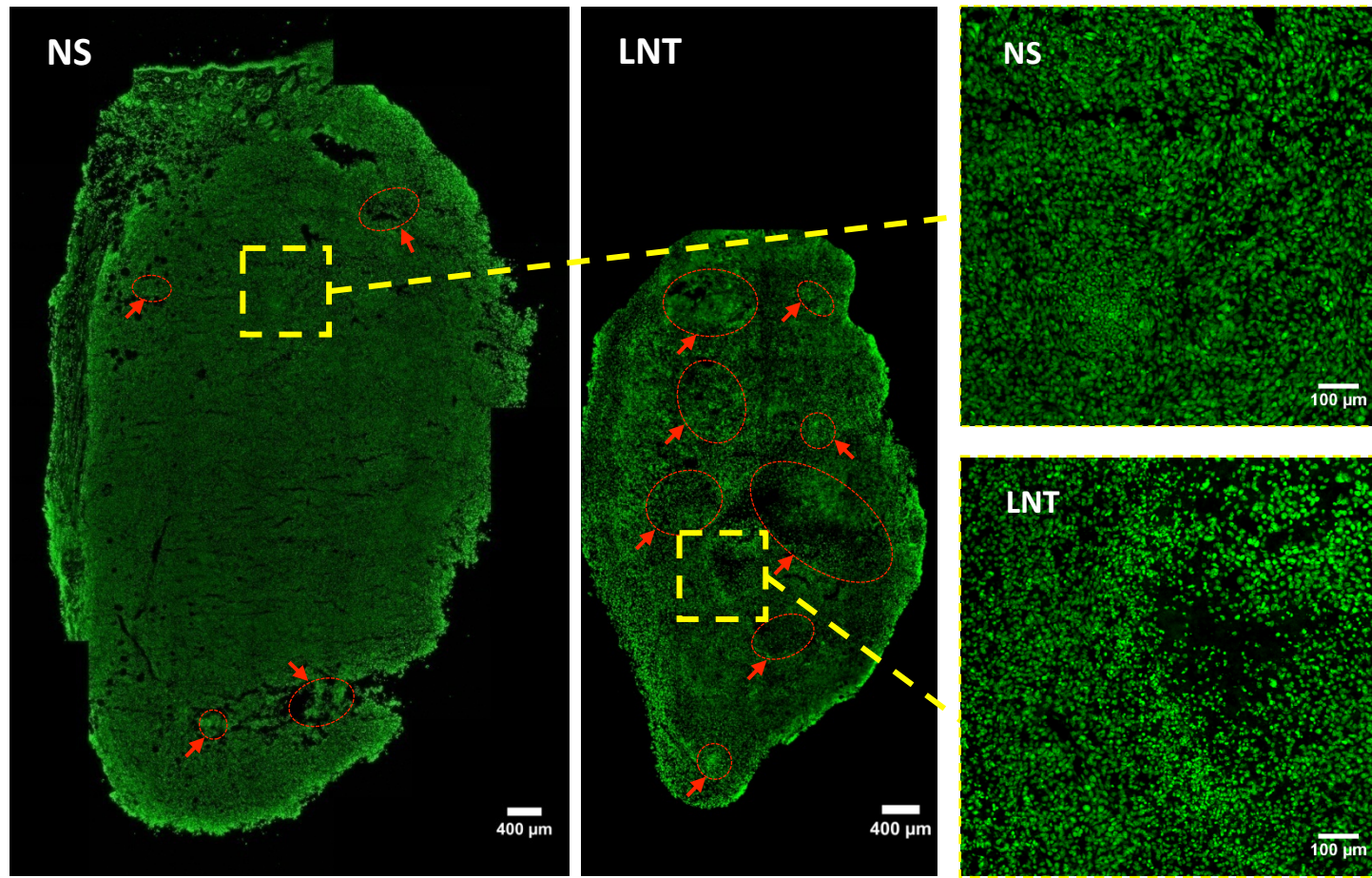

Green: Sytox green

Supplement: Supplementary file 1 — Methods: CT26 colorectal carcinoma cells (4x10^5^) or LAP0297 lung carcinoma cells (2x10^5^) were s.c. injected on the right flank of BALB/c or FVB mice, respectively. When tumors reached 4×4 mm in diameter, mice were randomly divided into 2 groups and received i.p. injection of saline (NS) or 1.0 mg/kg of LNT daily for 10 days. At the end of the treatments, tumor tissues were isolated, sectioned, and stained by an anti-CD31 antibody (endothelial cells). The proportions of endothelial cell area (CD31, Red) and Hoechst 33342 (Blue) perfused area over total tumor tissue area were quantified. SYTOX Green (Green) was used to label total cells. All data in the Additional file 1 are presented as means ± SEM. * p<0.05, ** p<0.01, *** p<0.001, and “ns” designates no significant difference. Fig. S1 LNT treatments inhibit tumor growth of CT26 colorectal carcinoma. a The experimental design of CT26 colorectal tumor model. b The growth curves and weight of CT26 colon tumors treated with saline or LNT. Fig. S2 LNT treatments do not affect the vasculature in normal colon tissues in LAP0297 tumor-bearing mice. Fig. S3 LNT treatments increase necrotic area. LAP0297 tumor tissues were sectioned and SYTOX Green (Green) was used to label tumor tissue cells. Necrotic cells were relatively small, dense, and round. Fig. S4 LNT treatments do not affect endothelial cell tube formation in vitro. HUVECs were seeded on the top of growth factor reduced matrigel in a 24-well plate, and then incubated with different concentrations of LNT. The process of tube formation was monitored every 3 hrs and pictures were taken at 12 hrs after incubation with LNT. The tubes and branches in each well were counted. Fig. S5 LNT treatments up-regulate the transcription of angiostatic factors, such as Ifnγ, Tnfα, Cxcl9, Ang1, Timp1, and Tsp1, in LAP0297 tumor tissues. Fig. S6 LNT treatments increase the expression of angiostatic factors, such as IFNγ, TNFα, CXCL9, Ang1, TIMP1, and TSP1, in LAP0297 tumor tissues. Fi [file 13046_2018_932_MOESM1_ESM.zip › Figure S3.pdf]

**Fig. S4**

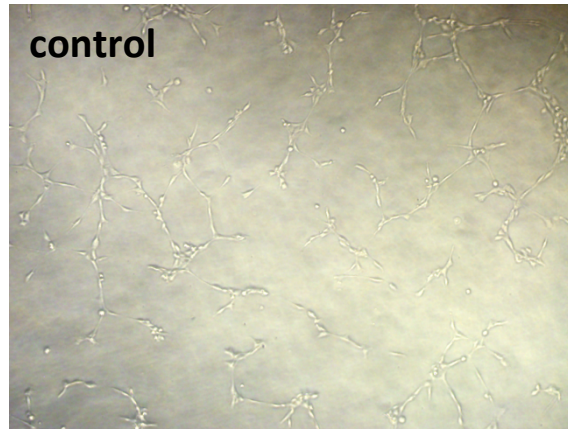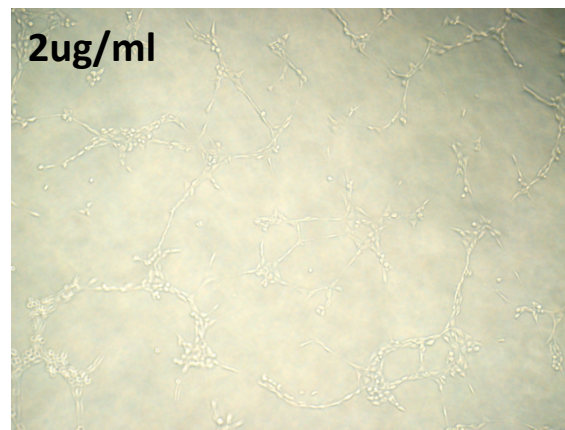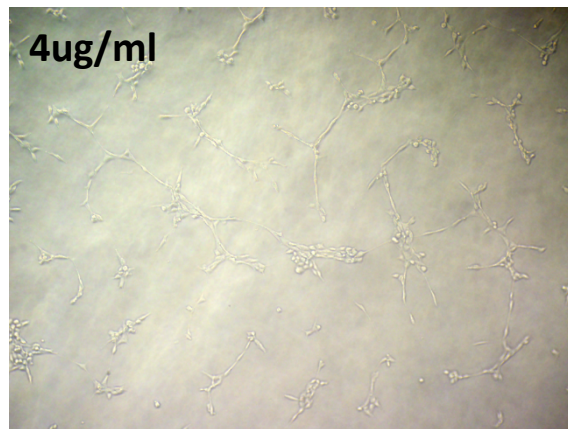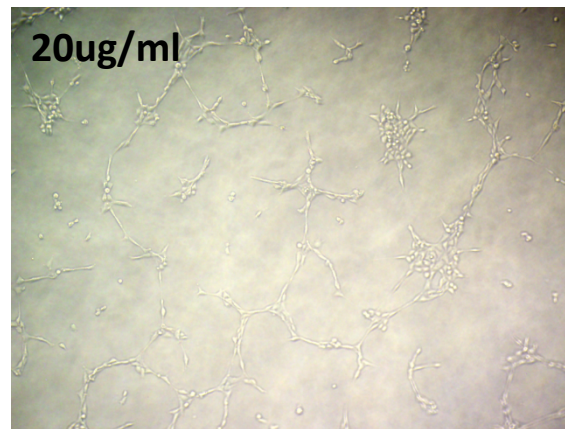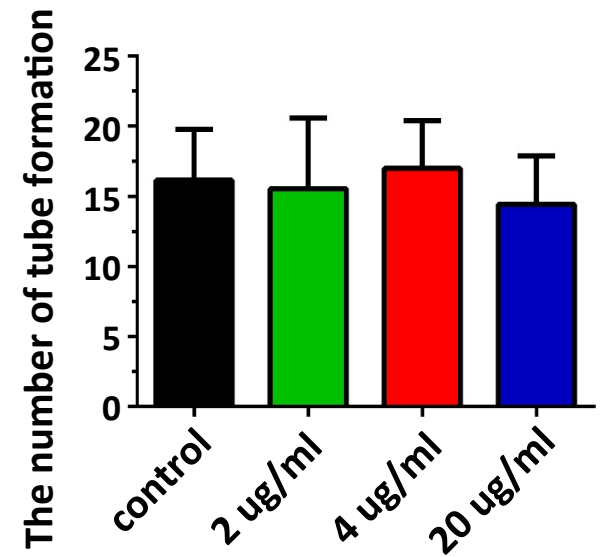

**The different concentrations of  
LNT treatments**

Supplement: Supplementary file 1 — Methods: CT26 colorectal carcinoma cells (4x10^5^) or LAP0297 lung carcinoma cells (2x10^5^) were s.c. injected on the right flank of BALB/c or FVB mice, respectively. When tumors reached 4×4 mm in diameter, mice were randomly divided into 2 groups and received i.p. injection of saline (NS) or 1.0 mg/kg of LNT daily for 10 days. At the end of the treatments, tumor tissues were isolated, sectioned, and stained by an anti-CD31 antibody (endothelial cells). The proportions of endothelial cell area (CD31, Red) and Hoechst 33342 (Blue) perfused area over total tumor tissue area were quantified. SYTOX Green (Green) was used to label total cells. All data in the Additional file 1 are presented as means ± SEM. * p<0.05, ** p<0.01, *** p<0.001, and “ns” designates no significant difference. Fig. S1 LNT treatments inhibit tumor growth of CT26 colorectal carcinoma. a The experimental design of CT26 colorectal tumor model. b The growth curves and weight of CT26 colon tumors treated with saline or LNT. Fig. S2 LNT treatments do not affect the vasculature in normal colon tissues in LAP0297 tumor-bearing mice. Fig. S3 LNT treatments increase necrotic area. LAP0297 tumor tissues were sectioned and SYTOX Green (Green) was used to label tumor tissue cells. Necrotic cells were relatively small, dense, and round. Fig. S4 LNT treatments do not affect endothelial cell tube formation in vitro. HUVECs were seeded on the top of growth factor reduced matrigel in a 24-well plate, and then incubated with different concentrations of LNT. The process of tube formation was monitored every 3 hrs and pictures were taken at 12 hrs after incubation with LNT. The tubes and branches in each well were counted. Fig. S5 LNT treatments up-regulate the transcription of angiostatic factors, such as Ifnγ, Tnfα, Cxcl9, Ang1, Timp1, and Tsp1, in LAP0297 tumor tissues. Fig. S6 LNT treatments increase the expression of angiostatic factors, such as IFNγ, TNFα, CXCL9, Ang1, TIMP1, and TSP1, in LAP0297 tumor tissues. Fi [file 13046_2018_932_MOESM1_ESM.zip › Figure S4.pdf]

Fig. S5

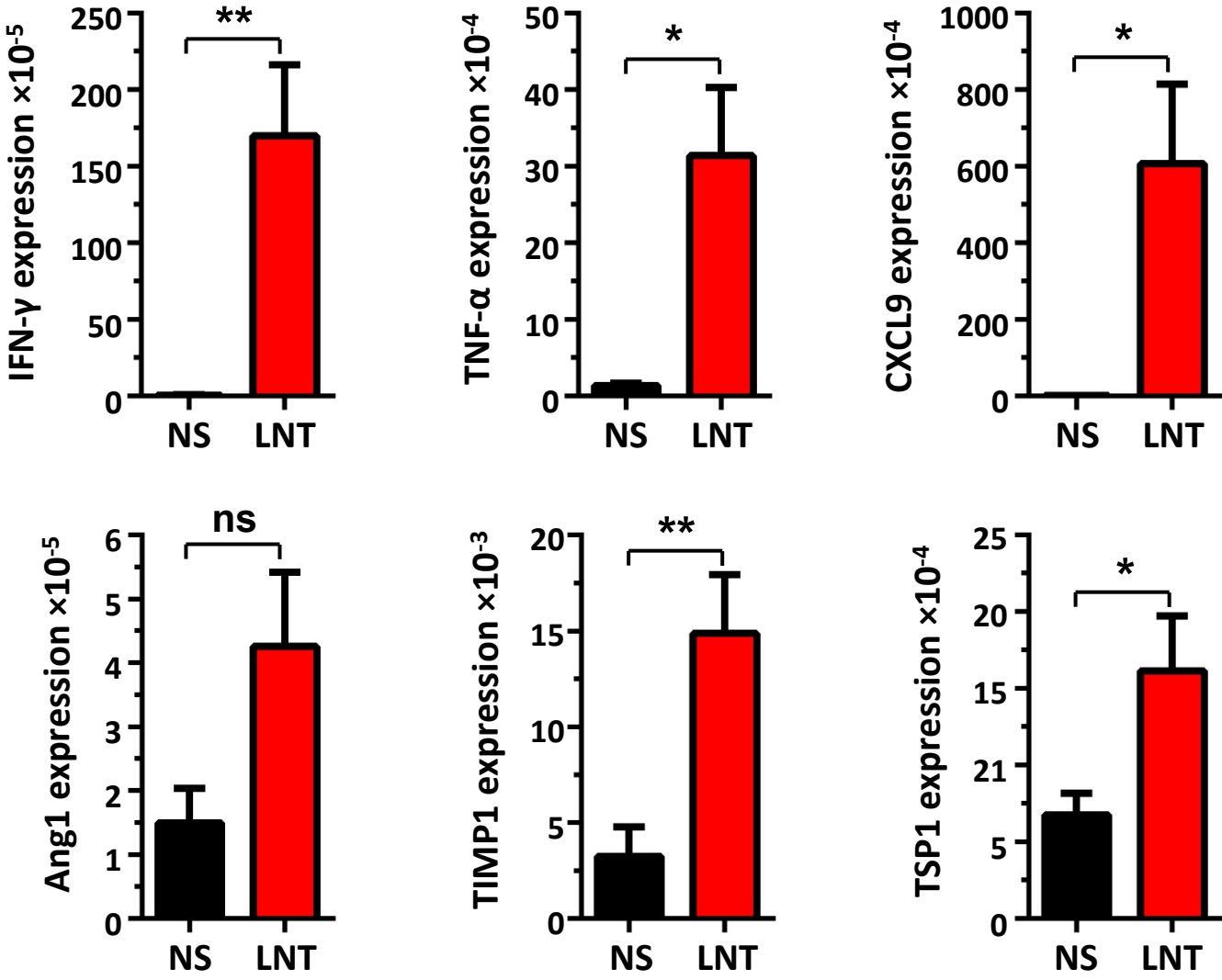

Supplement: Supplementary file 1 — Methods: CT26 colorectal carcinoma cells (4x10^5^) or LAP0297 lung carcinoma cells (2x10^5^) were s.c. injected on the right flank of BALB/c or FVB mice, respectively. When tumors reached 4×4 mm in diameter, mice were randomly divided into 2 groups and received i.p. injection of saline (NS) or 1.0 mg/kg of LNT daily for 10 days. At the end of the treatments, tumor tissues were isolated, sectioned, and stained by an anti-CD31 antibody (endothelial cells). The proportions of endothelial cell area (CD31, Red) and Hoechst 33342 (Blue) perfused area over total tumor tissue area were quantified. SYTOX Green (Green) was used to label total cells. All data in the Additional file 1 are presented as means ± SEM. * p<0.05, ** p<0.01, *** p<0.001, and “ns” designates no significant difference. Fig. S1 LNT treatments inhibit tumor growth of CT26 colorectal carcinoma. a The experimental design of CT26 colorectal tumor model. b The growth curves and weight of CT26 colon tumors treated with saline or LNT. Fig. S2 LNT treatments do not affect the vasculature in normal colon tissues in LAP0297 tumor-bearing mice. Fig. S3 LNT treatments increase necrotic area. LAP0297 tumor tissues were sectioned and SYTOX Green (Green) was used to label tumor tissue cells. Necrotic cells were relatively small, dense, and round. Fig. S4 LNT treatments do not affect endothelial cell tube formation in vitro. HUVECs were seeded on the top of growth factor reduced matrigel in a 24-well plate, and then incubated with different concentrations of LNT. The process of tube formation was monitored every 3 hrs and pictures were taken at 12 hrs after incubation with LNT. The tubes and branches in each well were counted. Fig. S5 LNT treatments up-regulate the transcription of angiostatic factors, such as Ifnγ, Tnfα, Cxcl9, Ang1, Timp1, and Tsp1, in LAP0297 tumor tissues. Fig. S6 LNT treatments increase the expression of angiostatic factors, such as IFNγ, TNFα, CXCL9, Ang1, TIMP1, and TSP1, in LAP0297 tumor tissues. Fi [file 13046_2018_932_MOESM1_ESM.zip › Figure S5.pdf]

**Fig. S6**

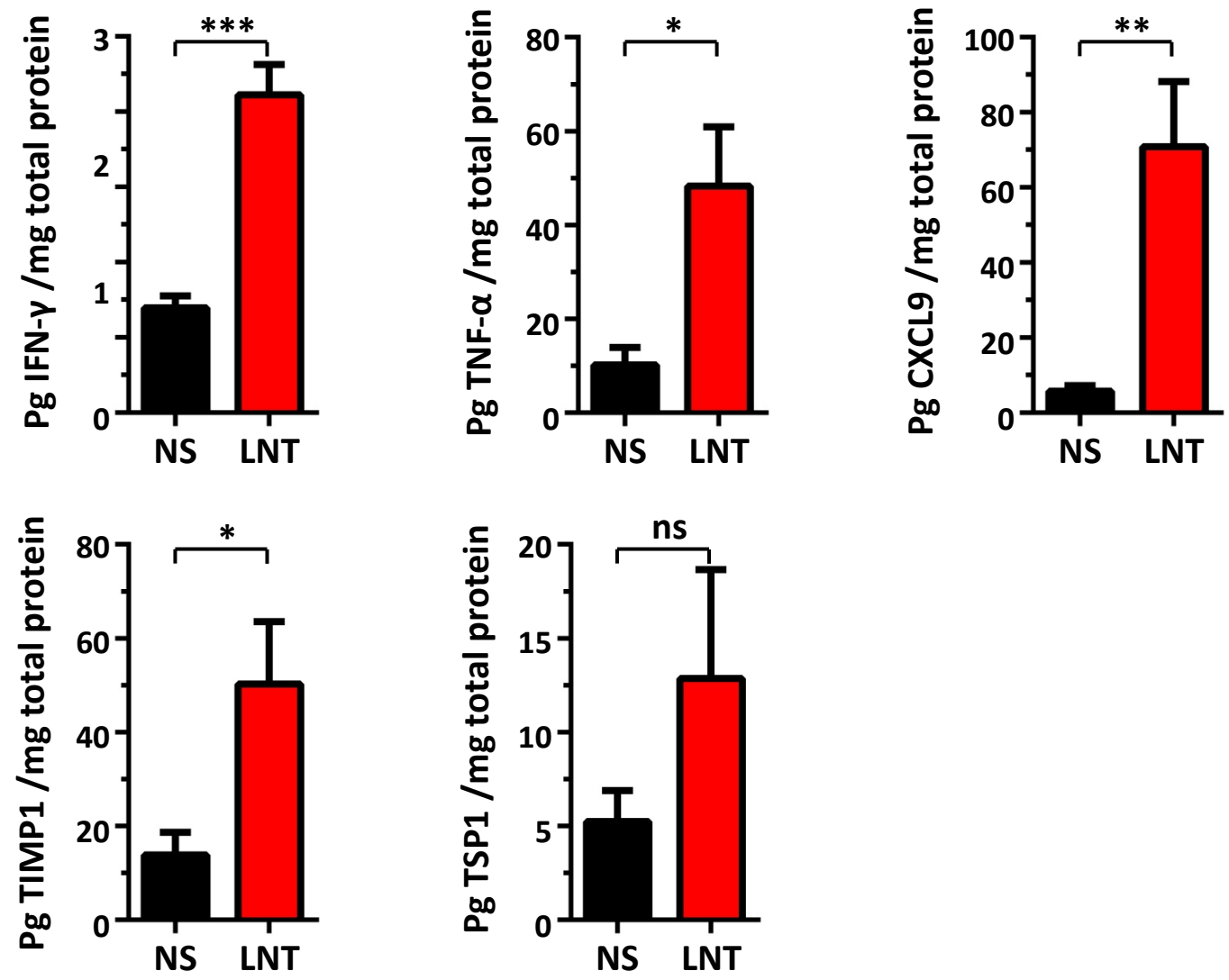

Supplement: Supplementary file 1 — Methods: CT26 colorectal carcinoma cells (4x10^5^) or LAP0297 lung carcinoma cells (2x10^5^) were s.c. injected on the right flank of BALB/c or FVB mice, respectively. When tumors reached 4×4 mm in diameter, mice were randomly divided into 2 groups and received i.p. injection of saline (NS) or 1.0 mg/kg of LNT daily for 10 days. At the end of the treatments, tumor tissues were isolated, sectioned, and stained by an anti-CD31 antibody (endothelial cells). The proportions of endothelial cell area (CD31, Red) and Hoechst 33342 (Blue) perfused area over total tumor tissue area were quantified. SYTOX Green (Green) was used to label total cells. All data in the Additional file 1 are presented as means ± SEM. * p<0.05, ** p<0.01, *** p<0.001, and “ns” designates no significant difference. Fig. S1 LNT treatments inhibit tumor growth of CT26 colorectal carcinoma. a The experimental design of CT26 colorectal tumor model. b The growth curves and weight of CT26 colon tumors treated with saline or LNT. Fig. S2 LNT treatments do not affect the vasculature in normal colon tissues in LAP0297 tumor-bearing mice. Fig. S3 LNT treatments increase necrotic area. LAP0297 tumor tissues were sectioned and SYTOX Green (Green) was used to label tumor tissue cells. Necrotic cells were relatively small, dense, and round. Fig. S4 LNT treatments do not affect endothelial cell tube formation in vitro. HUVECs were seeded on the top of growth factor reduced matrigel in a 24-well plate, and then incubated with different concentrations of LNT. The process of tube formation was monitored every 3 hrs and pictures were taken at 12 hrs after incubation with LNT. The tubes and branches in each well were counted. Fig. S5 LNT treatments up-regulate the transcription of angiostatic factors, such as Ifnγ, Tnfα, Cxcl9, Ang1, Timp1, and Tsp1, in LAP0297 tumor tissues. Fig. S6 LNT treatments increase the expression of angiostatic factors, such as IFNγ, TNFα, CXCL9, Ang1, TIMP1, and TSP1, in LAP0297 tumor tissues. Fi [file 13046_2018_932_MOESM1_ESM.zip › Figure S6.pdf]

Fig. S7

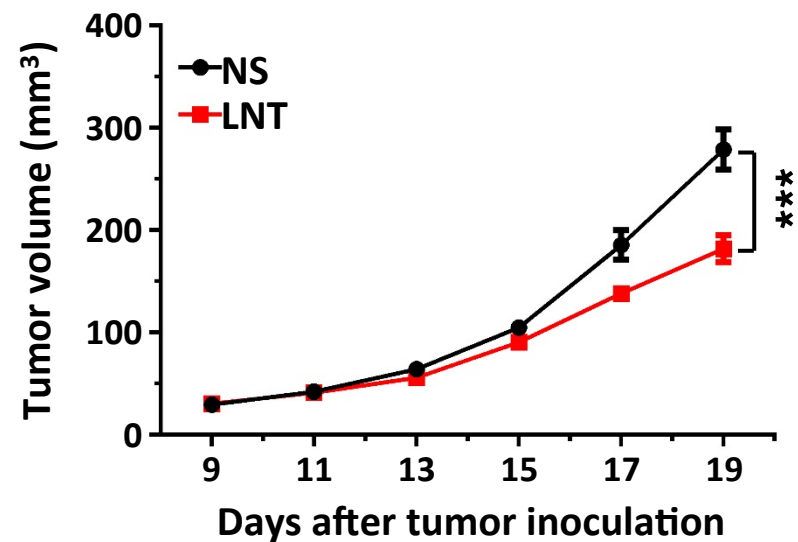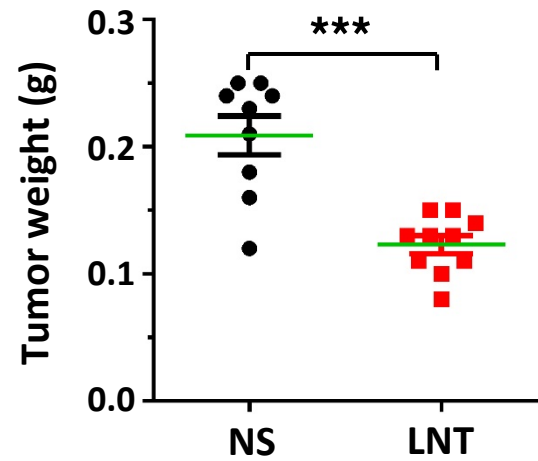

Supplement: Supplementary file 1 — Methods: CT26 colorectal carcinoma cells (4x10^5^) or LAP0297 lung carcinoma cells (2x10^5^) were s.c. injected on the right flank of BALB/c or FVB mice, respectively. When tumors reached 4×4 mm in diameter, mice were randomly divided into 2 groups and received i.p. injection of saline (NS) or 1.0 mg/kg of LNT daily for 10 days. At the end of the treatments, tumor tissues were isolated, sectioned, and stained by an anti-CD31 antibody (endothelial cells). The proportions of endothelial cell area (CD31, Red) and Hoechst 33342 (Blue) perfused area over total tumor tissue area were quantified. SYTOX Green (Green) was used to label total cells. All data in the Additional file 1 are presented as means ± SEM. * p<0.05, ** p<0.01, *** p<0.001, and “ns” designates no significant difference. Fig. S1 LNT treatments inhibit tumor growth of CT26 colorectal carcinoma. a The experimental design of CT26 colorectal tumor model. b The growth curves and weight of CT26 colon tumors treated with saline or LNT. Fig. S2 LNT treatments do not affect the vasculature in normal colon tissues in LAP0297 tumor-bearing mice. Fig. S3 LNT treatments increase necrotic area. LAP0297 tumor tissues were sectioned and SYTOX Green (Green) was used to label tumor tissue cells. Necrotic cells were relatively small, dense, and round. Fig. S4 LNT treatments do not affect endothelial cell tube formation in vitro. HUVECs were seeded on the top of growth factor reduced matrigel in a 24-well plate, and then incubated with different concentrations of LNT. The process of tube formation was monitored every 3 hrs and pictures were taken at 12 hrs after incubation with LNT. The tubes and branches in each well were counted. Fig. S5 LNT treatments up-regulate the transcription of angiostatic factors, such as Ifnγ, Tnfα, Cxcl9, Ang1, Timp1, and Tsp1, in LAP0297 tumor tissues. Fig. S6 LNT treatments increase the expression of angiostatic factors, such as IFNγ, TNFα, CXCL9, Ang1, TIMP1, and TSP1, in LAP0297 tumor tissues. Fi [file 13046_2018_932_MOESM1_ESM.zip › Figure S7.pdf]

Fig. S8

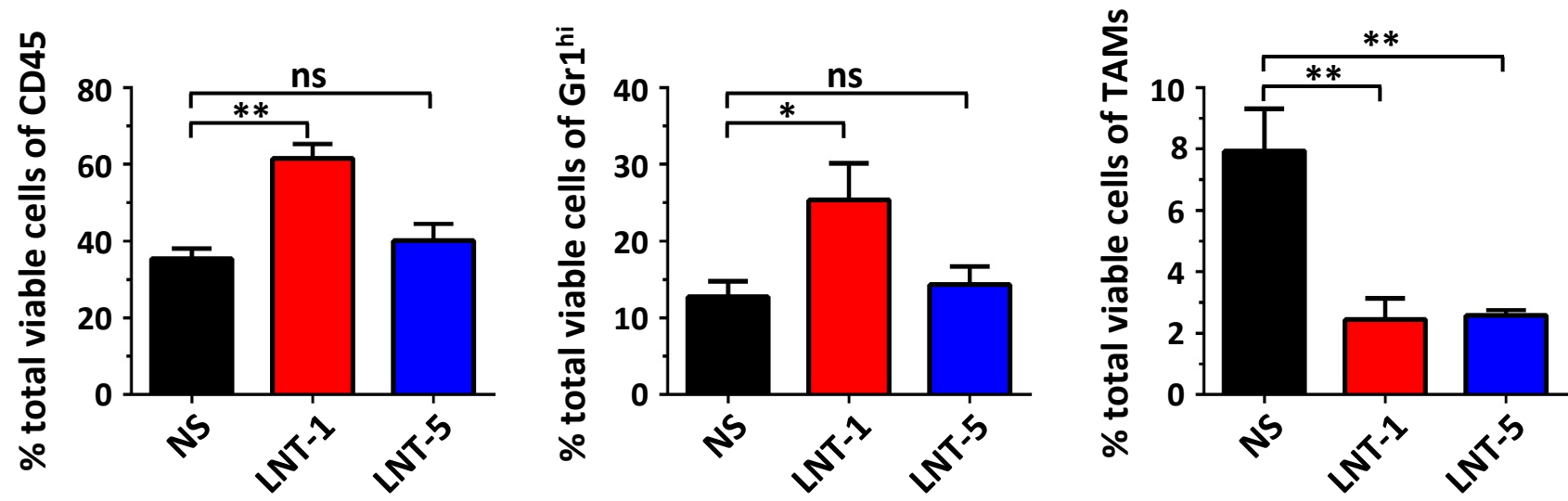

Supplement: Supplementary file 1 — Methods: CT26 colorectal carcinoma cells (4x10^5^) or LAP0297 lung carcinoma cells (2x10^5^) were s.c. injected on the right flank of BALB/c or FVB mice, respectively. When tumors reached 4×4 mm in diameter, mice were randomly divided into 2 groups and received i.p. injection of saline (NS) or 1.0 mg/kg of LNT daily for 10 days. At the end of the treatments, tumor tissues were isolated, sectioned, and stained by an anti-CD31 antibody (endothelial cells). The proportions of endothelial cell area (CD31, Red) and Hoechst 33342 (Blue) perfused area over total tumor tissue area were quantified. SYTOX Green (Green) was used to label total cells. All data in the Additional file 1 are presented as means ± SEM. * p<0.05, ** p<0.01, *** p<0.001, and “ns” designates no significant difference. Fig. S1 LNT treatments inhibit tumor growth of CT26 colorectal carcinoma. a The experimental design of CT26 colorectal tumor model. b The growth curves and weight of CT26 colon tumors treated with saline or LNT. Fig. S2 LNT treatments do not affect the vasculature in normal colon tissues in LAP0297 tumor-bearing mice. Fig. S3 LNT treatments increase necrotic area. LAP0297 tumor tissues were sectioned and SYTOX Green (Green) was used to label tumor tissue cells. Necrotic cells were relatively small, dense, and round. Fig. S4 LNT treatments do not affect endothelial cell tube formation in vitro. HUVECs were seeded on the top of growth factor reduced matrigel in a 24-well plate, and then incubated with different concentrations of LNT. The process of tube formation was monitored every 3 hrs and pictures were taken at 12 hrs after incubation with LNT. The tubes and branches in each well were counted. Fig. S5 LNT treatments up-regulate the transcription of angiostatic factors, such as Ifnγ, Tnfα, Cxcl9, Ang1, Timp1, and Tsp1, in LAP0297 tumor tissues. Fig. S6 LNT treatments increase the expression of angiostatic factors, such as IFNγ, TNFα, CXCL9, Ang1, TIMP1, and TSP1, in LAP0297 tumor tissues. Fi [file 13046_2018_932_MOESM1_ESM.zip › Figure S8.pdf]
